# Multimodality Imaging Leading the Way to a Prompt Diagnosis and Management of Transthyretin Amyloidosis

**DOI:** 10.3390/jcm14103547

**Published:** 2025-05-19

**Authors:** Anca Bălinișteanu, Roxana Cristina Rimbaș, Alina Ioana Nicula, Diana Piroiu, Adrian Dumitru, Amalia Ene, Dragoș Vinereanu

**Affiliations:** 1 Cardiology Department, University Emergency Hospital, 050098 Bucharest, Romania; 2 Carol Davila University of Medicine and Pharmacy, 050474 Bucharest, Romania; 3 Radiology Department, University Emergency Hospital, 050098 Bucharest, Romania; 4 Anatomo-Pathology Department, University Emergency Hospital, 050098 Bucharest, Romania; 5 Neurology Department, University Emergency Hospital, 050098 Bucharest, Romania

**Keywords:** transthyretin amyloidosis, cardiac amyloidosis, ATTR cardiomyopathy, multimodality imaging, COVID-19

## Abstract

**Background/Objectives:** A 43-year-old male presented with neurological symptoms and asymptomatic cardiac dysfunction, left ventricular hypertrophy, and impaired global longitudinal strain with apical sparing, associated with elevated NT-proBNP. **Methods:** Multimodality imaging (bone scintigraphy and cardiac magnetic resonance) revealed cardiac amyloid deposition. Genetic testing confirmed variant transthyretin amyloidosis (ATTR) with mixed phenotype. **Results:** Treatment with tafamidis 20 mg for stage I polyneuropathy, available at that moment, was initiated with good neurological outcome. Three years later, cardiac function deteriorated, following a moderate COVID-19 infection, with heart failure symptoms and reduced ventricular and atrial functions. For progressive ATTR cardiomyopathy, we intensified therapy to tafamidis free acid 61 mg, associated with SGLT2 inhibitor, spironolactone, and furosemide with subsequent improvements of symptoms and stabilization of imaging findings. **Conclusions:** This case emphasizes the importance of multimodal imaging in early detection, monitoring, and guiding individualized management in ATTR cardiomyopathy.

## 1. Introduction

Transthyretin amyloid cardiomyopathy (ATTR-CM) is a progressive infiltrative disorder resulting from the extracellular deposition of misfolded transthyretin amyloid fibrils in the myocardium. This process leads to increased myocardial stiffness, diastolic dysfunction, and ultimately progressive heart failure, often with poor prognosis and substantial healthcare burden [1,2]. Historically underdiagnosed due to its nonspecific presentation and clinical overlap with other forms of cardiomyopathy, ATTR-CM is now increasingly recognized as a potentially treatable condition [1]. Recent advances have introduced disease-modifying therapies, including transthyretin stabilizers and gene-silencing agents, that can alter the course of ATTR-CM, particularly when initiated in the early stages [1]. As a result, timely diagnosis has become crucial, prompting a paradigm shift in the diagnostic approach, risk stratification, and management strategies for these patients, emphasizing the need for heightened clinical suspicion, multimodal imaging, and timely intervention [2].

This case illustrates the complexity of diagnosing and managing variant ATTR-CM with mixed phenotype in a young, asymptomatic male presenting with early neurological symptoms and subtle cardiac findings. It underscores the indispensable role of multimodal imaging in early detection and monitoring, highlights the potential impact of COVID-19 as a trigger for cardiac disease progression, and emphasizes the need for individualized therapeutic strategies, including tailored therapeutic escalation, with tafamidis dose adjustment based on the clinical course.

## 2. Case Presentation

### 2.1. Patient Background

A 43-year-old male was referred to our cardiology department for evaluation in 2018. The patient complained of dizziness and painful paresthesia in the lower limbs, in the last 6 months prior to evaluation, being diagnosed with small fiber neuropathy and carpal tunnel syndrome. He practiced sportive efforts. His mother had longstanding heart failure with preserved ejection fraction, atrioventricular block with cardiac pacing. Physical examination did not reveal pathological signs.

### 2.2. Initial Investigation

**Figure 1:**
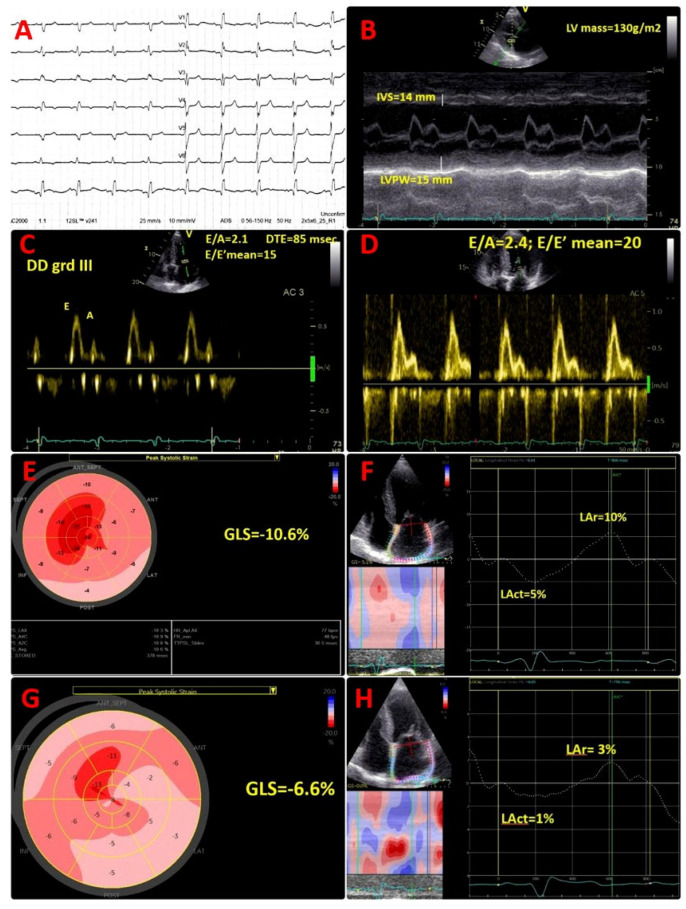
ECG and transthoracic echocardiographic images. (**A**) ECG- sinus rhythm, right bundle branch block, and pseudo-infarct pattern in V1–V3; (**B**) concentric left ventricular (LV) hypertrophy (IVS, interventricular septum of 14 mm, LVPW, LV posterior wall of 15 mm, LV mass of 130 g/m^2^); (**C**) restrictive filling pattern, with E/A of 2.1, DTE of 85 msec, E/E′ mean of 15, E = peak of early filling velocity, A = peak of late atrial filling velocity, DTE = mitral flow deceleration time, E′ = early diastolic tissue velocity; (**D**) worsening of diastolic dysfunction, restrictive filling pattern, with E/A of 2.4, E/E′ mean of 20; (**E**) bull’s eye plot by 2D speckle tracking echocardiography (STE) with significantly reduced global longitudinal strain (GLS) (−10.6%) and global altered deformation mainly at the basal and mid-ventricular segments, with a typical “apical sparing” pattern; (**F**) 2D STE at the level of the left atrium (LA) showing significantly decreased LA contractile function (Lact) and also of LA reservoir function (LAr); (**G**) bull’s eye plot by 2D speckle tracking echocardiography (STE) with additional significant reduction in global longitudinal strain (GLS) (−6.6%) and global altered deformation mainly at the basal and mid-ventricular segments, with a typical “apical sparing” pattern; (**H**) 2D STE at the level of the left atrium (LA) showing additional significantly reduction in LA contractile function (Lact = 1%) and also in LA reservoir function (Lar = 3%).

Cardiac troponin I was within the normal range; however, the N-terminal pro B-type natriuretic peptide (NT-proBNP) value was high at 687 pg/mL, in the absence of symptoms. Electrocardiogram (ECG) showed sinus rhythm, right bundle branch block, and pseudo-infarct pattern in V1–V3 (Figure 1A).

Transthoracic echocardiogram (TTE) described a diffuse granular sparkling pattern of the myocardium, with concentric left ventricular (LV) hypertrophy (interventricular septum of 14 mm, LV posterior wall of 15 mm, LV mass of 130 g/m^2^) and preserved LV ejection fraction (LVEF) of 52% (Figure 1B; Movie S1 in the Supplementary Material). Diastolic function assessment revealed restrictive pattern (grade III diastolic dysfunction), with E/A of 2.1, DTE of 85 msec, E/E′ mean of 15, low tissue Doppler LV myocardial velocities (septal E′ of 5 cm/s, lateral E′ of 9 cm/s), and severe left atrial enlargement (LAVi = 62 mL/m^2^) (Figure 1C). Of note, an abnormal global longitudinal strain (GLS = −10.6%), with apical sparring pattern was present (Figure 1E), associated with severely impaired left atrial reservoir and conduit strain (LAr = 10%, Lact = 5%) (Figure 1F). Moreover, TTE showed moderate tricuspid regurgitation, normal systolic pulmonary artery pressure (sPAP) = 25 mmHg, infiltrated thickened right ventricular (RV) free wall of 8 mm, reduced tricuspid plane systolic excursion (TAPSE) of 16 mm, and reduced longitudinal myocardial velocity (S’ of 8 cm/s).

### 2.3. Diagnostic Work-Up for Cardiac Amyloidosis

The association of newly diagnosed asymptomatic hypertrophic cardiomyopathy in a young patient, with a disproportionate NT-proBNP value, ECG features of pseudo-infarct pattern, and neurological involvement, raised a high index of suspicion for cardiac amyloidosis (CA).

**Figure 2:**
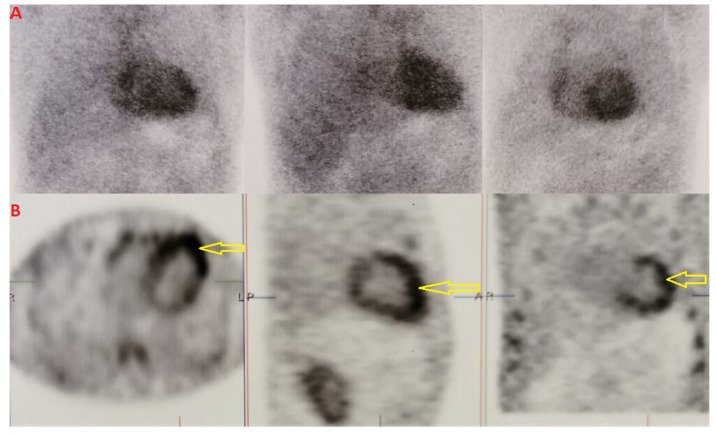
Bone scintigraphy with ^99m^Tc-DPD and single-photon emission computerized tomography (SPECT) imaging. (**A**) Bone scintigraphy with positive result defined as grade 3 = high myocardial uptake, greater than the bone. (**B**) SPECT confirming intra-myocardial uptake in both right and left ventricles (yellow arrows).

**Figure 3:**
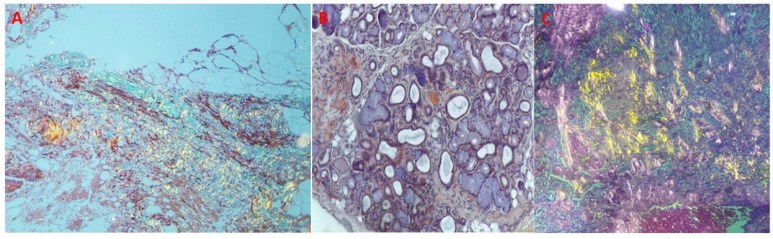
Anatomo-pathological evaluation: salivary gland and abdominal fat pad biopsies. (**A**) Amyloid deposits in adipose tissue showing yellow-green birefringence in polarized light—Congo red stain, polarized light microscopy 4×. (**B**) Small peri-glandular and perivascular amyloid deposits in a minor salivary gland. Congo red stain, 4×. (**C**) Amyloid deposits showing typical yellow-green birefringence in polarized light—Congo red stain, polarized light microscopy 4×.

We performed scintigraphy with ^99m^Tc-DPD and single photon emission computed tomography (SPECT) imaging, which showed grade 3 Perugini and confirmed biventricular myocardial uptake (Figure 2A,B). Furthermore, assessment for monoclonal proteins by serum and urine protein electrophoresis with immunofixation, and quantification of serum free-light chains revealed normal results. The patient underwent salivary gland and abdominal fat pad biopsies (internal protocol in 2018). The histological results were both positive for amyloid depositions (Figure 3A–C).

**Figure 4:**
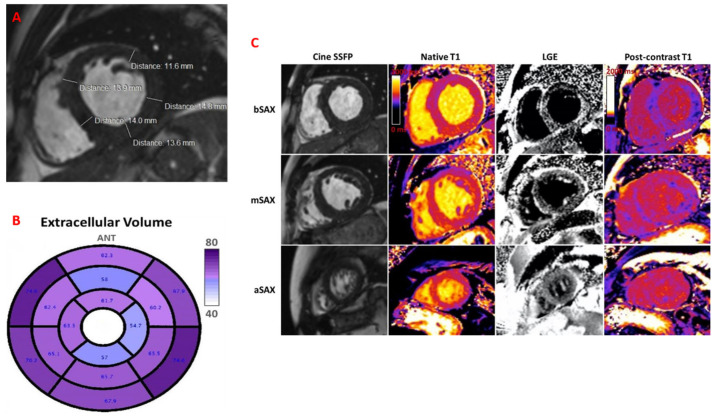
Cardiac magnetic resonance images. (**A**) Short-axis view showing left ventricular hypertrophy (IVS = 13.9 mm, AWT = 13 mm, LWT = 14.8 mm, PWT = 14 mm). (**B**) Extracellular volume (ECV) bull’s eye plot evaluated by cardiac magnetic resonance, using a 16-segment model, displaying a progressive decrease of the ECV from base to apex, explaining the substrate of apical sparing deformation pattern. (**C**) Short axis views at basal (bSAX), midventricular (mSAX) and apical (aSAX) levels showing concentric left ventricle hypertrophy in SSFP cine images (the first column), with diffuse elevation in native T1 mapping (the second column), transmural hyperenhancement, more pronounced at the basal and midventricular levels, with a dark blood pool in LGE images (the third column) and diffuse reduction in post-contrast T1 mapping (forth column). SSFP: Steady-state free precession; LGE: late gadolinium enhancement.

Cardiac magnetic resonance (CMR) revealed LV hypertrophy with LVEF of 45%, non-dilated ventricles, and significantly elevated native T1 mapping (mean value of 1436 ms) and extracellular volume (ECV) of 62–76% (Figure 4A–C). Genetic testing confirmed variant ATTR and detected sequence variants: p.E109k(p89k), c325G>A. A similar mutation was found in his mother’s genotype.

### 2.4. Diagnosis

The patient qualified for diagnosis of variant ATTR with mixed phenotype: stage I polyneuropathy and preclinical cardiomyopathy.

### 2.5. Initial Treatment

In accordance with the National Program for TTR amyloidosis, at that moment, the patient was started on specific therapy with tafamidis 20 mg daily, for stage I polyneuropathy. The patient showed good tolerance to treatment, without further peripheral neurologic impairment, with similar cardiac evaluation, yearly.

### 2.6. Follow-Up

However, in 2021, at the 3-year follow-up visit, regardless of the favourable clinical neurological response with similar electromyography findings, the patient showed accelerated cardiac disease progression, begun after a moderate COVID-19 infection.

He presented with dyspnoea on moderate exertion and fatigue, NYHA class II, bilateral leg oedema with significantly increased NT-proBNP value of 3368 pg/mL. Echocardiographic assessment showed deterioration of LV systolic function, with reduced LVEF (39%) and decreased GLS (−6.6%), and worsening of diastolic function parameters: mean E/E′ ratio of 20, lower tissue Doppler LV diastolic myocardial velocities (septal E′ of 4 cm/s, lateral E′ of 5 cm/s). An additional decline of LA reservoir and conduit strain (LAr = 3%, Lact = 1%) (Figure 1D,G,H) was found, suggestive for progressive atrial myopathy. A similar progression of RV dysfunction was detected, with decreased TAPSE of 14 mm. ECG monitoring (24 h) did not show any arrhythmias. Transoesophageal echocardiography showed no thrombus.

### 2.7. Therapeutic Adjustment and Outcomes

At this point, the patient was started on a short course of intravenous diuretic therapy, followed by oral diuretic medication (furosemide and spironolactone). We also decided to initiate dapagliflozine 10 mg. The patient did not tolerate ARNI and β-blocker, due to hypotension. Taking into account the rapidly evolving ATTR-CM, we decided on transition from tafamidis 20 mg daily to tafamidis free acid 61 mg daily.

Follow-up at every 6-month intervals revealed favourable clinical outcomes, without HF symptoms, and with similar findings on the echocardiographic assessment, with significantly decreased NTproBNP value. Regular cardiologic follow-up with cardiac biomarkers, ECG and echocardiography, is mandatory for optimal care. CMR at 2 years demonstrated LVEF of 45% and a mean ECV of 70%, almost similar to the previous one, suggestive of non-progressive disease.

## 3. Discussion

The particularity of our case was the accelerated cardiac impairment, after COVID-19 infection, in a patient with mixed ATTR phenotype, under a disease-modifying therapeutic drug for stage I polyneuropathy. The reported one-year mortality of hereditary ATTR is 20% [1]. The cornerstone of therapy in ATTR-CM is prompt diagnosis. Hence, our case highlighted the pivotal role of multimodality imaging in the early recognition of cardiac involvement in variant ATTR and also in the follow-up plan, timely and accurate diagnosis being essential for guiding an individualized disease-modifying approach [2].

Several conditions can present with hypertrophic phenotypes that mimic hypertrophic cardiomyopathy (HCM), including cardiac amyloidosis. CMR plays a key role in distinguishing true HCM from its phenocopies by providing detailed tissue characterization, allowing for the assessment of myocardial fibrosis, and extracellular volume analysis [3]. These parameters yield both diagnostic and prognostic insights, providing more accurate classification and guiding individualized treatment strategies [3].

Moreover, although not routinely included in the standard diagnostic work-up for cardiac amyloidosis, myocardial work has emerged as a novel tool for assessing myocardial function, particularly in hypertrophic phenotypes. As demonstrated by de Gregorio et al., myocardial work indices are significantly more impaired in patients with ATTR-CM compared to those with hypertrophic cardiomyopathy, highlighting its potential utility in differentiating these conditions [4].

Recent evidence highlights the potential role of cardiopulmonary exercise testing (CPET) in the early detection, functional assessment and risk stratification of cardiac amyloidosis [5]. By identifying subtle hemodynamic impairments before clinical manifestations arise, CPET may enhance risk stratification when used alongside conventional tools [5]. Nonetheless, further studies are needed to establish its standardized use in this clinical setting.

COVID-19 infection may trigger the new onset of HF due to the direct effect of the virus on the myocardium or the increased sympathetic drive, and systemic inflammatory mediators leading to myocardial depression, and subsequently to LV dysfunction, as in our case [6].

Patients with pre-existing chronic cardiovascular conditions are known to be at an elevated risk of severe complications and increased mortality when infected with COVID-19 [7]. However, there remains a significant gap in the literature regarding the specific impact of COVID-19 on patients with rare cardiac conditions, such as ATTR cardiomyopathy [7]. Given the progressive nature of ATTR-CM, which is characterized by myocardial infiltration, diastolic dysfunction, and conduction abnormalities, even mild systemic stressors have the potential to exacerbate cardiac impairment [2].

A retrospective observational study conducted by Larrañaga-Moreira et al. provided valuable insights into the clinical outcomes of patients with cardiac amyloidosis and COVID-19 infection [8]. The study found that these patients exhibited disproportionately higher rates of hospitalization and mortality compared to the general population, exceeding what would be expected based on their advanced age and sex [8].

These findings highlight the critical need for further research to characterize the long-term cardiovascular sequelae of COVID-19 in patients with ATTR-CM. Future prospective studies should aim to determine whether COVID-19 infection accelerates amyloid progression or leads to an irreversible decline in cardiac function. Moreover, the role of early therapeutic interventions, including heart failure management and intensified amyloid-targeted therapies, could be essential in improving outcomes and preventing rapid disease progression in this vulnerable patient group.

Despite the availability of treatment, disease progression can still occur, emphasizing the necessity of therapeutic escalation strategies. The decision to increase the tafamidis dosage to 61 mg was driven by the patient’s deteriorating cardiac function and supported by clinical trial evidence. Notably, the observed stabilization following dose adjustment suggests that early up-titration may be beneficial in selected cases, potentially slowing disease progression and enhancing clinical outcomes.

The new HF guidelines for the diagnosis and treatment of acute and chronic heart failure clearly stated that tafamidis is recommended as Class I indication for patient with NYHAII class HF with ATTR cardiomyopathy [9]. Tafamidis, at both 80 and 20 mg, has been proved to lower all-cause mortality and cardiovascular-related hospitalizations, in patients with ATTR cardiomyopathy [1]. However, in terms of efficacy of each dose, an important reduction in risk of death was demonstrated with tafamidis meglumine 80 mg daily (equivalent to tafamidis free acid 61 mg), compared with 20 mg daily [10]. Therefore, we decided to upgrade the treatment to tafamidis 61 mg.

Asymptomatic individuals with a confirmed pathogenic mutation associated with variant transthyretin amyloidosis should undergo regular assessment to detect early signs of cardiac involvement. Current recommendations suggest performing a standard ECG annually and 24 h ambulatory ECG monitoring every six months, to monitor for the development of arrhythmias or conduction abnormalities that may signal early cardiac disease [11].

In cases of cardiac amyloidosis, particularly variant ATTR, a multidisciplinary approach is essential for accurate diagnosis, risk stratification, and personalized management. As highlighted in recent expert recommendations, such patients benefit from assessment by a dedicated cardiomyopathy team within specialized centers, where multimodal imaging, genetic counselling, and tailored therapeutic strategies can be optimally integrated [12].

## 4. Conclusions

In summary, a comprehensive clinical and multimodal imaging approach are mandatory for early diagnosis and individualized management of patients with ATTR.

Timely identification of cardiac involvement, even in the absence of overt symptoms, allows for the initiation of disease-modifying therapies at a stage when they are most effective, thus improving outcomes.

## Data Availability

The original contributions presented in this study are included in the article/Supplementary Material. Further inquiries can be directed to the corresponding author.

## Supplementary Materials

The following supporting information can be downloaded at: https://www.mdpi.com/article/10.3390/jcm14103547/s1. Movie S1. Transthoracic echocardiography-apical 4 chamber view. Granular sparkling of the interventricular septum, concentric left ventricular hypertrophy, left ventricular systolic dysfunction, and thickening of mitral and tricuspid valve leaflets.
